# Testing job wellbeing indicators among community behavioral health workers: Community-based participatory research

**DOI:** 10.1371/journal.pone.0321351

**Published:** 2025-04-23

**Authors:** Sadaaki Fukui, Wei Wu, Jennifer Garabrant, Michelle P. Salyers, Nathaniel Dell, Emily Bass, Jaime Greenfield, Gary Morse

**Affiliations:** 1 School of Social Work, Indiana University, Indianapolis, Indiana, United States of America; 2 Department of Psychology, Indiana University Indianapolis, Indiana, United States of America; 3 Department of Psychiatry, Washington University School of Medicine, St. Louis, Missouri, United States of America; 4 BJC Healthcare, St. Louis, Missouri, United States of America; 5 Places for People, Inc., St. Louis, Missouri, United States of America.; Universiti Pertahanan Nasional Malaysia, MALAYSIA

## Abstract

Many community behavioral health organizations (CBHOs) continue to struggle with their employees’ reduced job wellbeing and job disengagement (i.e., turnover intentions, actual turnover). Understanding employees’ job wellbeing priorities in their organizational contexts is essential to address the challenges, especially for workers in diverse work settings such as CBHOs. We used community-based participatory research (CBPR) strategies to develop and test job wellbeing indicators. The current study implemented 11 indicators with 168 people employed at a CBHO through initial and 6-month follow-up surveys. Positive endorsement of job wellbeing indicators differed based on employees’ demographic (e.g., race, education, marital status) and job (e.g., exempt status, clinical positions) characteristics. Several indicators declined from the initial to the follow-up surveys (e.g., communication, job fairness, decision-making involvement, expectation alignment, supervisory support, career development opportunities). The change rates also varied by employee characteristics (e.g., work years, race, exempt status, full-time). The current study illustrates the utility of CBPR strategies to implement job wellbeing indicators based on employees’ priorities and diverse job wellbeing experiences among employee subpopulations. Further, the developed indicators revealed job wellbeing heterogeneity by employee subpopulations within an organization that is often overlooked. Efforts to understand varying job wellbeing characteristics among diverse employees may eventually help develop organization-tailored interventions to improve job wellbeing and reduce turnover.

## Introduction

Excessive turnover has been a significant problem for many community behavioral health organizations (CBHOs), with reported annual turnover rates ranging from 25% to 60% [1–3]. Turnover negatively impacts the organization, individual workers [1,3–6], and the quality of care for clients [3,5,7,8]. High turnover can be indicative of more pervasive turnover intention among employees remaining with the organization. Supporting workers’ job wellbeing priorities is imperative for meeting the growing demand for quality and accessible behavioral health care.

Burnout is a significant factor that impedes job wellbeing among the population [9,10]. Behavioral health systems have continued to be underfunded [11,12](e.g., low wages, shortage of the workforce, increased caseloads, high job demands) while behavioral health workers work with clients in highly challenging situations (e.g., severe symptoms, low income, no insurance, unstable living conditions), leading to increased job demands and turnover [13]. Organizational researchers have tried to understand and address the challenges by applying existing theories and models such as the job demands-resources model [14](understanding both job demands and resources).

Although burnout and job wellbeing have been historically discussed more at the individual level, increased attention to organizational factors is suggested in behavioral health [15,16]. According to Leiter and Maslach [17], organizational factors that may contribute to burnout and job wellbeing involve six areas, inclusive of (1) increased workload; (2) limited control/autonomy; (3) insufficient/inconsistent reward with workers’ expectations; (4) diminished sense of community; (5) unfairness; and (6) value conflicts on the job.

These organizational factors have been studied in burnout and turnover research in behavioral health. For instance, risk factors include insufficient salary, lack of organizational support, lack of professional development opportunities [18,19], job demands [20], emotional labor burden, low organizational trust [18], workplace interpersonal conflict, and heavy workload [21]. Conversely, protective factors include positive workplace climates, fair performance appraisal, job autonomy, and workplace psychological safety [8].

Despite knowledge of general risk and protective factors related to job wellbeing, CBHOs continue to struggle with these challenges. CBHOs are complex, comprised of workers representing varying characteristics (e.g., demographics, job roles, perceptions). CBHOs typically offer heterogeneous programs (e.g., outpatient, medication prescription, residential care, home-based program, school-based program, therapy, peer support, specialized evidence-based programs) within the organization for varying populations (e.g., populations with mental health difficulties, substance use, other cooccurring disorders, population ages may range greatly from children, youth, adults, to older adults) in different geographic locations (e.g., urban, suburban, rural). Accordingly, the local CBHO’s employee characteristics may not reflect the characteristics of the samples in the research literature.

Many existing studies use cross-sectional surveys testing standard job wellbeing scales (e.g., burnout, job satisfaction, work-life balance, work autonomy) which limit the number of target constructs to avoid a lengthy survey. Job wellbeing variations by employee subpopulations (e.g., employee and job characteristics) within an organization are not often the analytical targets, although their job wellbeing priorities can be more fluid than static as organizational contexts change. Akin to measurement-based care [22], it is important to understand workers’ job wellbeing priorities based on their characteristics within their organizational contexts. To achieve this, we conducted community-based participatory research (CBPR) to develop indicators to measure job wellbeing that reflect the contexts of the implementation organization and to test them. Below, we will illustrate the CBPR approaches used to develop the job wellbeing indicators, which will be tested in the current study.

## Community-Based Participatory Research (CBPR) to develop job wellbeing indicators

We employed CBPR strategies in developing job wellbeing indicators at a CBHO. CBPR is a collaborative research approach, involving concerned stakeholders and researchers, to integrate their knowledge and prompt action to address emerging issues in the community [23]. CBPR features stakeholders’ engagement in research processes, co-identification of research questions, collaborative implementation of research tasks, and co-dissemination of knowledge and actions based on established partnerships [23]. CBPR is a useful approach for addressing gaps in translational research knowledge [24], particularly for addressing growing concerns and interests in disparities among diverse communities [23]. As such, CBPR is a suitable approach for developing and testing job wellbeing indicators as a bottom-up person-centered approach (vs. the traditional top-down organization-centered management approach [25]) to address the long-lasting challenges.

Our CBPR approach involved employees of the organization (from frontline workers to senior leadership) at all levels of the study, including designing the study, interpreting results, and writing manuscripts. The collaborative efforts began with the leadership’s questions, including why employees leave, who needs support, and how to support employees to promote retention and growth. These questions led to the need to understand employees’ priorities, characteristics, and status of job wellbeing. Specifically, research questions originated by the organization included: (1) are there any individual differences in job wellbeing by employees’ characteristics? and (2) are there any individual differences in change of job wellbeing by employees’ characteristics? The goal was to develop brief job wellbeing indicators and explore the use that can be informative for organization-tailored job wellbeing interventions. We applied existing (yet often unused) strategies to develop and implement job wellbeing indicators to understand employees’ job wellbeing characteristics. These strategies included job exit surveys, stay interviews, and workgroups for job wellbeing measurement development [26]. The study can provide an insight into the potential job wellbeing variations based on employees’ characteristics and the importance of identifying job wellbeing priorities through CBPR for tailored interventions.

## CBPR implementation setting

The CBPR site was a CBHO that employed approximately 300 employees in an urban area in the Midwestern United States, which served approximately 2,500 clients annually. The CBHO provides evidence-based interventions to support recovery among people with severe mental illness and substance use disorders, including assertive community treatment, supported employment, medication management, and other psychosocial approaches.

## CBPR implementation goal and process

The goal of the CBPR was to collaborate with stakeholder workgroups, consisting of CBHO employees, to develop critical job wellbeing indicators to understand the characteristics among employees and to monitor them for quality improvement on an ongoing basis (e.g., periodical organization-wide survey, employee evaluation, supervision). Job wellbeing is a multifaceted construct; however, research has shown the usefulness of single-item job wellbeing measures to reducing survey-response burden while increasing face or content validity with content experts’ input [27,28]. To maximize the construct coverage of single-item measures, it is important to develop and evaluate the measures within the implementation contexts [27], especially, given the challenges to capture the heterogeneity presence in job wellbeing constructs (e.g., based on job roles, workers, and workplace) [29]. Jarden & Siegert (29) suggest a need for instrument development that involves stakeholders throughout the development and testing phases. Note that our aim was different from a regular job wellbeing scale development with multi-item scales to capture a latent construct. Rather, we intended to use the indicators individually to reflect different job wellbeing priorities of the organization for identifying target intervention areas on a regular basis without added survey-response burden. The study was planned and funded prior to the COVID-19 pandemic and our inquiry was formed as a general inquiry related to job wellbeing (i.e., not conducting specific inquiries related to the COVID-19 pandemic).

## Job wellbeing indicator development through CBPR

In order to identify job wellbeing priorities, we employed exit surveys and stay interviews. We then used the findings to develop job wellbeing indicators through workgroups and roundtable meetings. Although some study results have been reported elsewhere, it is necessary to include them for a complete portrayal of the CBPR process. These results are briefly summarized below to minimize redundancy.

## Exit survey

Exit surveys/interviews of people leaving the organization can be useful for understanding job disengagement patterns [30–33]. We utilized online Qualtrics exit surveys at the CBHO for those who notified the Human Resources (HR) department about their resignation (i.e., leavers). The survey questions were developed by the research team and the organization’s employees, including what made them stay until deciding to leave and the most critical reason for leaving. The exit surveys were sent out to 61 leavers (between 16/03/2020 and 26/10/2021), and 35 responded (58% response rate). The respondents included 74% female, 60% White, and 34% married, and their average age was 37.2 (SD =10), and average work years in the organization was 3.8 (SD = 3.8). We used content analysis to identify emerging themes [34,35] with feedback provided by the organization’s staff. Five major turnover themes were identified: (1) *struggles in current job roles*, (2) *negative experiences with upper management*, (3) *quality of care concerns*, (4) *no foreseeable future*, and (5) *personal/family reasons* [36].

## Stay interview

The research team emailed 23 employees who had stayed at the CBHO for at least 14 years for stay interviews in 2020 (between 15/07/2020 and 23/11/2020). Thirteen employees (56.5%) participated in Zoom-based individual interviews. They were 55.7 (SD = 10.3) years old on average, worked in the organization for an average of 20.8 (SD = 4.3) years, 54% were females, 85% were White, and 54% were married. We asked the interviewees why they believe they have stayed at the organization so long, if there were ever times when they considered leaving (and what helped them stay), and why they thought others have stayed or left the organization. We followed an emergent content analytic approach [37] with elements of immersion and crystallization [38]. Four main themes were identified: (1) *work as a calling*, (2) *supportive relationships*, (3) *opportunities for growth or meaningful contribution*, and (4) *personal attributes or values* [39].

## Job wellbeing indicator formation

Results of the exit surveys and stay interviews were synthesized by the research team and the organization’s employees to salient features that may impact employees’ job wellbeing and turnover. The research team then conducted two-stage, 1-hour workgroup sessions with the organization’s employees to develop job wellbeing indicators. We obtained a list of employees and their email addresses from the HR department. We recruited a total of 14 employees (between 20/07/2021 and 31/08/2021), and 4 participated in both workgroup stages; 9 employees participated in each stage [Female: N=12 (86%); White: N=13 (93%)].

The first stage involved validation or member-checking [40] of the themes identified through the exit surveys and stay interviews and a discussion of the employees’ priority for each theme. In August 2021, the participants reviewed the identified themes presented during the first-stage workgroup session (See online supplement S1 Fig. Synthesized Themes of Exit Surveys and Stay Interviews). The major themes were *people*, *flexibility/autonomy*, *fairness/inclusiveness*, *misfit*, *importance of routine/job stability*, *feeling valued, no better place*, *opportunities for growth*, t*he organization/team/work climate*, *support*, and *workload*. The participants were asked to provide feedback, including the overall impressions of the identified themes, check agreement, any missing themes or comprehensiveness, and priority for the organization. Based on the feedback from the first stage workgroup participants, the research team and the organization’s staff developed a draft of the job wellbeing indicators using the themes agreed upon by the participants. Initially, 22 items were generated based on the participants’ statements from the exit surveys, stay interviews, and the first stage workgroup (See online supplement S2 Fig. Prioritized Job Wellbeing Concerns).

In September 2021, the research team invited the second stage of the workgroup participants. The participants reviewed the synthesized themes of the exit surveys and stay interviews and the 22 items to assess whether the priorities were adequately reflected. The workgroup participants also assessed the items’ importance, comprehensiveness, and wording. For instance, the workgroup suggested that “my supervisor knows me as a person” was vague. This led to an alternative wording: “I feel lucky to have my supervisor who supports a good quality of work and job wellbeing.”

Feedback was also brought to the organization’s leadership through routinely scheduled meetings and written summaries, and the research and leadership teams evaluated the 22 items with the goal of selecting 10 critical job wellbeing indicators, avoiding a lengthy survey. The wording of items was also evaluated. For example, the organization’s staff and the research team suggested that the supervision item should be separated into two: one for supervisory support for employees’ job wellbeing and the other for the quality of care (e.g., a supervisor may care about job quality but may not necessarily address job wellbeing). We finalized the job wellbeing indicators with 11 items (See Table 1) with face validity. The importance of the 11 items was also affirmed during the CBPR implementation by a total of 48 employees who attended onsite and virtual roundtable meetings (the CBHO sent the invitation to all employees) in June 2022 (between 09/06/2022 and 14/06/2022).

**Table 1:** Job Wellbeing Indicators (11 items).

	Never	Rarely	Sometimes	Often	Very Often	Always	Not Applicable
Communication from upper management is clear.	1	2	3	4	5	6	NA
People are included in decision-making.	1	2	3	4	5	6	NA
Employees are treated fairly.	1	2	3	4	5	6	NA
My expectations align well with the expectations of management.	1	2	3	4	5	6	NA
My degree pays off.	1	2	3	4	5	6	NA
I have opportunities to advance my career here.	1	2	3	4	5	6	NA
My work is flexible to accommodate my personal/family needs.	1	2	3	4	5	6	NA
My supervisor cares about my well-being.	1	2	3	4	5	6	NA
My supervisor supports the quality of my work.	1	2	3	4	5	6	NA
I am acknowledged and my input is valued here.	1	2	3	4	5	6	NA
Stress overweighs positive feelings that may come from my work.	1	2	3	4	5	6	NA

Note: higher scores indicate better job wellbeing except for the stress indicator where a higher score indicates more stress.

With the developed job wellbeing indicators, we conducted organization-wide surveys twice (initial and 6-month follow-up surveys) to examine the job wellbeing status based on the employees’ demographics and job characteristics at the initial survey as well as their associations with changes in their job wellbeing status at the follow-up survey. Below, we detail the survey methods, data analyses, and results, followed by the discussion and implications of the main findings of the current study.

## Method

Study procedures were approved by the Indiana University Institutional Review Board. Informed consent was obtained from the participants, and waiver of informed consent was applied to the data already being collected by the organization (i.e., demographic variables, job/position characteristics). The participants were provided with the IRB-approved Informed Consent Statement and indicated in the online form if they agreed to participate or not. The data were de-identified (with research IDs) and cleaned by the research team for analysis.

We obtained active employees’ work email addresses from HR and sent all employees a confidential Qualtrics initial survey (with the 11 job wellbeing indicators) via email (between 27/10/2021 and 12/01/2022), and then a 6-month follow-up survey (between 10/08/2022 and 20/10/2022) to capture any changes since the initial survey. We added two survey questions to capture the employees’ perceptions about the changes in the follow-up survey. They included (*1) Have you noticed any efforts within the organization to address workplace well-being in the past 6 months?*(1. *Have not noticed any efforts* to 4. *Noticed substantial efforts*) and (*2) Have you noticed any changes in your or your colleagues’ workplace well-being in the past 6 months?*(1. *Substantially declined workplace well-being* to 5. *Substantially improved workplace well-being*).

We received 174 responses (response rate 51%, or 174/342), with 170 valid cases for the initial job wellbeing survey. For the follow-up survey, we received 170 responses (47% or 170/365 response rate), with 163 valid cases. Due to turnover and new hires during the six months, the employee pools were different between the two surveys. Because our interest was to track changes and capture their associated factors, we retained 168 cases who completed the initial survey for the analyses (Table 2). The cases who only participated in the follow-up survey were excluded, most of whom were newer hires. Out of the 168 cases, 100 (60%) participated in both surveys, 39 (23%) did not participate in the follow-up survey, and 29 (17%) left the organization by then.

**Table 2:** Job Wellbeing Survey Participants’ Demographics (N=168).

	N	%
*Gender*		
Female	127	75.6
Male	41	24.4
*Marital Status*		
Married	62	36.9
Single	106	63.1
*Employee Status*		
Full Time	145	86.3
Part Time	10	6.0
*Employee Type*		
Clinical	131	78.0
Non-clinical	37	22.0
*Race*		
White	116	69.0
Employee of Color (one Asian ottherwise Black)	51	31.0
*Exempt Status*		
Exempt	42	25.0
Non-Exempt	126	75.0
*Educational Degree*		
Bachelors or below	91	54.2
Masters or higher	77	45.8
*Position*		
Admin Assist/Assistant/coordinator	5	3.0
Direct Provider	48	28.6
Finance	7	4.2
Leadership	9	5.4
Nurse	13	7.7
Outreach/Community	10	6.0
Peer Provider	3	1.8
Residential Service	7	4.2
Team Leader/Manager	39	23.2
Therapist/Prescriber	17	10.1
Other	10	6.0
	***M***	***SD***
*Age*	43.8	13.0
*Work years at the organization*	5.2	5.8

## Data analysis

We first examined whether any of the employees’ demographics and job characteristics were associated with each job wellbeing outcome using the initial survey data. We conducted bivariate analyses. We then used latent change score models (LCSMs, see Castro-Schilo and Grimm [41] & McArdle [42]) in R lavaan [43] to examine job wellbeing changes which 1) accounted for measurement errors by treating change scores as latent variables, and 2) included cases with incomplete data using the full information maximum likelihood estimation method [44]. Two sets of models were tested. The first set examined the change in each job wellbeing outcome. The second set added a set of predictors simultaneously to examine whether they were associated with the change in each outcome.

The predictors were extracted from the available HR data, mirroring the previous research suggesting the predictability of demographics and job characteristics for turnover [45]. They include age, gender (Female=1 vs. Male=0), race (White=1 vs. People of Color=0, only one Asian otherwise Black), marital status (Married=1 vs. Single=0), educational degree (Master’s degree or higher=1 vs. Bachelor’s degree or below=0), work years at the organization, full time (Full Time=1 vs. Part Time=0), work type (Clinical=1 vs. Non-clinical), exempt status and (Exempt=1 vs. Non-Exempt=0: Exempt employees are salary-based employees, not hourly, thus they are not subject to overtime pay). Other predictors include organization’s workplace wellbeing effort notice and job wellbeing change notice.

We reported both significant (p ≤.05) and marginally (p ≤.10) significant effects, given the exploratory nature and the presence of potentially small sub-groups in our data. We also reported the effect sizes, including Cohen’s *d*s (denoted as *d*) and standardized partial regression coefficients (denoted as *β*), with *d* capturing a standardized difference between two group means (i.e., 0.2, 0.5, and 0.8 are small, medium, and large differences, respectively [46]), and *β* reflecting the magnitude of a unique association between two variables.

## Results

### Job wellbeing descriptive statistics and associated factors

The correlations among the 11 job wellbeing indicators can be found in Table 3, and the descriptive statistics are included in Table 4. Table 5 shows the significant or marginally significant results of the associated factors. White employees rated their involvement in decision-making processes (*d* =.4, *p* <.01) and clarity of communication (*d* =.4, *p* =.02) lower than employees of color, although employees of color rated supervisory support for the quality of work lower (*d* =.3, *p* =.063). Compared to non-clinical employees, clinical employees rated decision-making process involvement (*d* =.3, *p* =.09) and job fairness (*d* =.3, *p* =.10) lower. Employees who were not married rated job fairness lower (*d* =.3, *p* =.06) and perceived that their degree paid off less (*d* =.3, *p* =.10) compared to married employees. Employees with a Bachelor’s degree or below perceived that their degree paid off (*d* =.3, *p* =.07) less and reported less stress (*d* =.3, *p* =.09) than employees with a Master’s degree or higher. Non-exempt employees perceived that their degree paid off (*d* =.3, *p* =.09) less than exempt employees.

**Table 3:** Correlations among the 11 Job Wellbeing Indicators.

		1	2	3	4	5	6	7	8	9	10	11
1	Communication from upper management is clear	1.00										
2	People are included in decision-making	0.75	1.00									
3	Employees are treated fairly	0.61	0.63	1.00								
4	My expectations align well with the expectations of management	0.65	0.64	0.52	1.00							
5	My degree pays off	0.36	0.42	0.41	0.41	1.00						
6	I have opportunities to advance my career here	0.39	0.46	0.52	0.41	0.47	1.00					
7	My work is flexible to accommodate my personal/family needs	0.36	0.37	0.49	0.42	0.40	0.44	1.00				
8	My supervisor cares about my well-being	0.30	0.30	0.52	0.37	0.33	0.42	0.56	1.00			
9	My supervisor supports the quality of my work	0.42	0.34	0.48	0.50	0.41	0.44	0.55	0.84	1.00		
#	I am acknowledged and my input is valued here	0.43	0.51	0.57	0.54	0.39	0.56	0.47	0.70	0.75	1.00	
#	Stress overweighs positive feelings that may come from my work	0.30	0.23	0.33	0.33	0.33	0.29	0.33	0.30	0.24	0.27	1.00

Note: To align the directionality for easier interpretation, the 11^th^ indicator is reverse-coded, thus higher scores reflect lower stress in this correlation matrix

**Table 4:** Job Wellbeing Descriptive Statistics (Initial & 6-mo Follow-up), Change Results, and the Predictive Factor Effects from the Latent Change Score Models.

	*Initial*			*6-mo Follow-up*			*Change*	*Factors (with p* ≤ *.10)*
	*N*	*M*	*SD*	*N*	*M*	*SD*		
Communication from upper management is clear	164	3.7	1.2	100	3.4	1.1	Δ = −0.83, *d* = .69, *p* = .04	Work years (B = .05, *β* = 0.23, *p* = .04)
People are included in decision-making	166	3.2	1.2	100	3.0	1.2	Δ = −0.77, *d* = 0.67, *p* = .04	
Employees are treated fairly	168	4.1	1.1	100	3.8	1.1	Δ = −1.45, *d* = 1.37, *p* < .01	Notice change (B = .25, *β* = 0.27, *p* = .02)
My expectations align well with the expectations of management	164	4.0	1.2	93	3.8	1.1	Δ = −0.89, *d* = 0.75, *p* = .04	Work years (B = .05, *β* = 0.23, *p* = .04)
								Race (B = .47, *β* = 0.37, *p* = .10)
My degree pays off	142	3.8	1.5	86	3.8	1.5	No Sig.	Gender (B = −0.62, *β* = −0.42, *p* = .10)
I have opportunities to advance my career here	157	3.7	1.3	93	3.5	1.3	Δ = −0.97, *d* = 0.74, *p* = .06	Clinical employee (B = .77, *β* = 0.57, *p* = .02)
								Work years (B = .05, *β* = .20, *p* = .07)
My work is flexible to accommodate my personal/family needs	168	4.6	1.3	100	4.8	1.2	No Sig.	Notice change (B = .23, *β* = 0.24, *p* = .05)
My supervisor cares about my well-being	168	4.9	1.4	100	4.9	1.4	No Sig.	Work years (B = .05, *β* = 0.22, *p* = .05)
My supervisor supports the quality of my work	166	4.9	1.2	100	4.7	1.4	Δ = −1.11, *d* = 0.91, *p* = .02	Exempt (B = -.56, *β* = -.42, *p* = .10)
I am acknowledged and my input is valued here	166	4.2	1.4	98	3.9	1.3	No Sig.	Work years (B = .06, *β* = 0.26, *p* = .01)
								Exempt (B = −1.01, *β* = -.71, *p* < .01)
Stress overweighs positive feelings that may come from my work	166	3.0	1.1	99	3.4	1.1	Δ = 1.29, *d* = 1.15, *p* = .01	Full time (B = −1.86, *β* = −1.40, *p* = .02)
								Notice change (B = -.45, *β* = −36, *p* < .01)
Have you noticed any efforts within the organization to address workplace well-being in the past 6 months?	N/A			97	2.9	0.9	N/A	N/A
Have you noticed any changes in your or your colleagues’ workplace well-being in the past 6 months?	N/A			98	2.7	1.0	N/A	N/A

Note: *M* stands for observed mean. *SD* is standard deviation. *N* is sample size. Δ indicates the estimated latent mean difference between the first and second surveys. *d* is Cohen’s d, representing a standardized mean difference. *B* and *β* represent unstandardized and standardized regression coefficients, respectively.

**Table 5:** Factors associated with Job wellbeing Indicators.

	*Demographcs & Job Factors*		
Communication from upper management is clear	White Employee:	*M*=3.5 (1.2)	*t*(162)=2.42, *p*=.017
	Employee of Color:	*M*=4.0 (1.3)	
People are included in decision-making	Clinical Employee:	*M*=3.1(1.1)	*t*(164)=1.71, *p*=.089
	Non-Clinical:	*M*=3.5 (1.2)	
	White Employee:	*M*=3.1 (1.1)	*t*(164)=2.73, *p*=.007
	Employee of Color:	*M*=3.6 (1.3)	
Employees are treated fairly	Married:	*M*=4.3 (1.0)	*t*(166)=1.87, *p*=.063
	Single:	*M*=4.0 (1.1)	
	Clinical Employee:	*M*=4.1(1.0)	*t*(166)=1.66, *p*=.10
	Non-Clinical:	*M*=4.4 (1.3)	
My expectations align well with the expectations of management			
My degree pays off	Exempt:	*M*=4.1(1.3)	*t*(140)=−1.73, *p*=.085
	Non-Exempt	*M*=3.7(1.5)	
	Master's or above:	*M*=4.0 (1.4)	*t*(140)=−1.83, *p*=.069
	Bachelor's or below:	*M*=3.5(1.5)	
	Married:	*M*=4.1(1.4)	*t*(140)=−1.68, *p*=.10
	Single:	*M*=3.6 (1.5)	
I have opportunities to advance my career here			
My work is flexible to accommodate my personal/family needs			
My supervisor cares about my well-being			
My supervisor supports the quality of my work	White Employee:	*M*=5.1 (1.1)	*t*(74.1)=−1.89, *p*=.063
	Employee of Color:	*M* =4.6 (1.4)	
I am acknowledged and my input is valued here			
Stress overweighs positive feelings that may come from my work	Master's or above:	*M*=3.2 (1.0)	*t*(164)=−1.7, *p*=.09
	Bachelor's or below:	*M*=2.9 (1.2)	

Note: *M* indicates an observed group means, and t indicates the t-test for the group mean difference. The demographics tested included gender, marital status, full-time status, clinical, race, exempt status, and educational degree. The absolute correlation coefficients of the job wellbeing indicators with age and previous work years were < 0.15.

### Job wellbeing change

The results from LCSMs without predictors (Table 4) suggested a significant decrease in clarity of communication (*d* =.69, *p* =.04), job fairness (*d* = 1.37, *p* <.01), decision-making processes involvement (*d* = 0.67, *p* =.04), expectation alignment (*d* = 0.75, *p* =.04), and supervisory support for the quality of work (*d* = 0.91, *p* =.02), and a significant increase in job stress (*d* = 1.15, *p* =.01). In addition, there was a marginally significant decrease in carrier advancement opportunities (*d* = 0.74, *p* =.06).

### Predictors of job wellbeing change

The results from LCSMs with predictors (Table 4) suggested that holding the other predictors constant, work years at the organization was associated with the decrease of clarity of communication (*β* = 0.23, *p* =.04), expectation alignment (*β* = 0.23, *p* =.04), feel valued (*β* = 0.26, *p* =.01), or career advancement opportunities (*β* =.20, *p* =.07). On average, employees with longer work years showed less decrease in these outcomes. In addition, perceived improvement of workplace job wellbeing (who noticed changes) had a positive effect on the change of job fairness (*β* = 0.27, *p* =.02), job flexibility (*β* = 0.24, *p* =.05), and stress (*β* = −36, *p* <.01). Employees who noticed more changes in job wellbeing within the organization expressed a smaller decrease in job fairness and flexibility or a smaller increase in stress. Also, work type was related to the change in carrier advancement opportunities (*β* = 0.57, *p* =.02), with clinical employees perceiving a smaller decrease in the opportunities than non-clinical employees on average. In addition to the notice of the job wellbeing changes within the organization, full-time status was associated with the change in stress (*β* = 1.40, *p* =.02), with part-time employees expressing more increase in stress than full-time employees. In addition to work years, exempt employees showed a greater decrease in feeling of being valued. Finally, a few factors showed marginally significant effects on some outcomes. Employees of color expressed more decrease in expectation alignment than White employees (*β* = 0.37, *p* =.10), males revealed more decrease in their perception regarding the payoff of their degrees than females (*β* = −0.42, *p* =.10), and exempt employees exhibited more decrease in perceived supervisor’s support for the quality of work (*β* = -.42, *p* =.10).

## Discussion

Our CBPR approach synthesized lived experience and research expertise, which resulted in 11 job wellbeing indicators with face validity. The identified job wellbeing areas are consistent with the literature on behavioral health employee wellbeing [16]. They reasonably shared variances, yet also tapped unique job wellbeing aspects. Our study revealed varying job wellbeing characteristics across employee subpopulations within an organization. The findings suggest the importance of organization-based job wellbeing assessment and interventions.

Clinical employees, employees with lower educational degrees, and non-exempt employees tended to experience lower job wellbeing. Concerning perceived stress, employees with a Master’s degree or higher tended to perceive more stress than those with a Bachelor’s degree or below. Regarding marital status (unmarried/single employees tended to rate lower), it is important to consider other contextual factors, such that unmarried/single employees were significantly younger (*p* =.03) or worked at the organization for shorter years (p <.01), which might contribute to the differences.

Additionally, White employees rated their involvement in decision-making processes and clarity of communication from upper management lower compared to employees of color. Our findings appear contradictory based on recent research about racial disparities, negatively impacting employees of color [47]. However, when considering that employees of color rated their supervisory support for the quality of work lower compared to White employees, these findings may provide further insight that may coincide with the notion of structural racism.

Employees of color tend to occupy a greater proportion of positions in frontline roles (which are often lower in the organizational hierarchy) as a means of increasing the diversity of the frontline workforce to bear greater resemblance to the clientele served [47]. Consequently, minoritized employees may set lower expectations around communication with upper management or involvement in decision-making processes (e.g., feel less relevant, compared to White employees who might have more proximity to upper management). On the other hand, supervisory support for the quality of care may have a more direct impact on employees of color. Our stay interviews also found a similar trend: White employees tended to discuss their ability to impact organizational changes, while Black employees tended to relate their work passion back to their own community as their reason for staying [39].

Further, our findings revealed a significant decrease in clarity of communication, job fairness, decision-making process involvements, expectation alignment, supervisory support for the quality of work, career advancement opportunities, and an increase in job stress. It was a challenging time for the organization, experiencing the continuing negative impacts of the COVID-19 pandemic, including transitions of executive leadership and challenges to fill position vacancies. During the CBPR period, the CBHO developed several initiatives as part of their strategic plans, including increasing support to mitigate burnout, promoting diversity, equity, inclusion, and belonging, increasing wages and benefits for certain positions to keep up with the rising costs of inflation, facilitating professional development opportunities, improving work environment safety, and improving communication among staff. Indeed, in our indicators, 66% of the survey participants noticed either moderate or substantial organizational efforts to address workplace wellbeing. At the same time, 41% noticed no change in workplace wellbeing, and the remaining responses were divided into 37% who perceived declined wellbeing, and only 22% who perceived improved wellbeing. This illustrates the job wellbeing heterogeneity within an organization.

Finally, employees with longer work years or who noticed more changes in the organization’s efforts to address workplace wellbeing showed less decrease in some job wellbeing indicators. Employees with longer tenure may be more resilient or able to better work around job wellbeing, given their seniority within the organization (e.g., more job autonomy; [17]). Clinical employees perceived a smaller decline in career development opportunities. The CBHO invested its efforts in creating more career advancement opportunities for this subpopulation that might have contributed to the result. The increase in work stress among part-time employees could be due to the instability of income among this subpopulation, while exempt employees might experience a greater decrease in feeling valued due to an increase in work with a limited salary increase.

Some study limitations need to be noted. First, our findings were drawn from one CBHO which limits the generalizability. Second, we were not able to reach some employee subpopulations despite our significant efforts. For instance, in order to better engage with Black employees, we invited a Black social work researcher whose expertise was racial disparities, used researchers with the same racial background for interview participants, and consulted with an inclusion committee. Regardless, the participation rates of employees of color were disproportionate. Based on the organization’s HR data, 59% were White, and 41% were employees of color. However, 70% of those who completed the initial survey were White employees, and 78% of those who completed both surveys were White employees. Third, our study was implemented in an open and natural setting; thus, direct and indirect causes for the observed changes (or no changes) could not be examined. Examining the driving factors for change is a critical next step to facilitate a deeper understanding of the wider implications of the job wellbeing indicators beyond the current observational study. For such future efforts, our findings provide informative insights into what variables need to be included in the predictive modeling (e.g., control variables). Fourth, we were not able to look at the impact of job roles/positions on changes due to the small sample size for each category, especially given the significant variations within the same job category. Finally, we cannot isolate the potential historical confound of the pandemic. Interestingly, the pandemic was not frequently mentioned as a major theme in our exit surveys and interviews except for some (e.g., leaving the job to stay home with children, the pandemic shut down most other job opportunities for a while). However, recent studies discuss the potential negative impact of the pandemic on behavioral health employees’ wellbeing [48,49].

Our study contributions follow. First, the study suggests the utility of the community-academic partnership in leveraging multiple data sources and creating diverse ways for employees to provide input and participate in the process for implementing job wellbeing indicators. Our indicators can be used by other CBHOs; however, it requires careful assessment of their contexts and priorities, given that our study revealed varying job wellbeing characteristics and priorities (i.e., the heterogeneity among employee subpopulations for each element) even within an organization, not to mention across organizations. Our next step is to streamline and strategize our CBPR approaches (e.g., using technologies such as artificial intelligence, machine learning, mobile apps) to amplify community organizations’ capacities for job wellbeing indicator development and implementation, especially those with fewer resources. The job wellbeing indicators can be used during regular supervision sessions (except for supervision-related ones), periodical organization-wide checkups, and annual evaluations. Subsequently, the indicators should help develop organization-tailored interventions to improve job wellbeing and reduce turnover among diverse employees.

## Conclusion

Behavioral health organizations in the U.S. continue to struggle with employees’ high burnout and extensive turnover. Although existing theories and models (e.g., burnout theory, turnover theory, the job demands-resources model) have been applied in behavioral health research, the gaps have not been filled in our practice settings in part due to the limitations in literature (e.g., the population models may not be fully applied to the local contexts). Accordingly, it is essential to evaluate the priorities and experiences of employees in their work environments to build bottom-up approaches to inform employee-centered data-driven organizational decision-making for improving their employees’ job wellbeing in their organizational contexts. Using a community-based participatory research approach, the current study implemented the 11 job wellbeing indicators that were reflective of the involved employees’ priorities. Testing the indicators, including the potential driving causes for changes in future studies, will further enhance the utility.

## Supporting information

S1 Fig Synthesized Themes of Exit Surveys and Stay Interviews. (TIF)

S2 Fig Prioritized Job Wellbeing Concerns. (TIF)
