# Supplementary figures and images for: Testing job wellbeing indicators among community behavioral health workers: Community-based participatory research
Source: PLoS One. 2025 Apr 23;20(4):e0321351. doi: 10.1371/journal.pone.0321351 (PMC12017500; doi:10.1371/journal.pone.0321351)

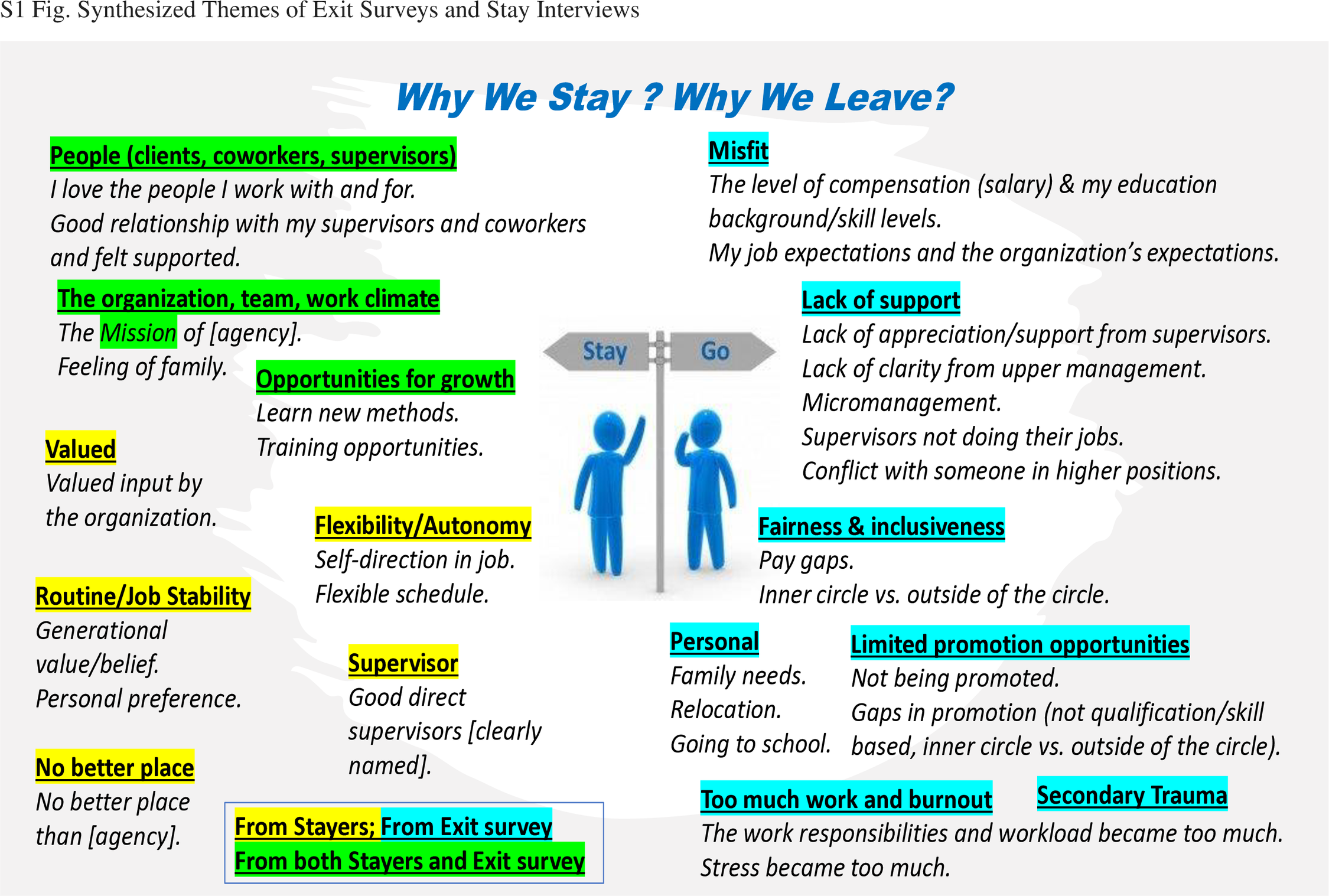

Supplement: S1 Fig — (TIF) [file pone.0321351.s001.tif]

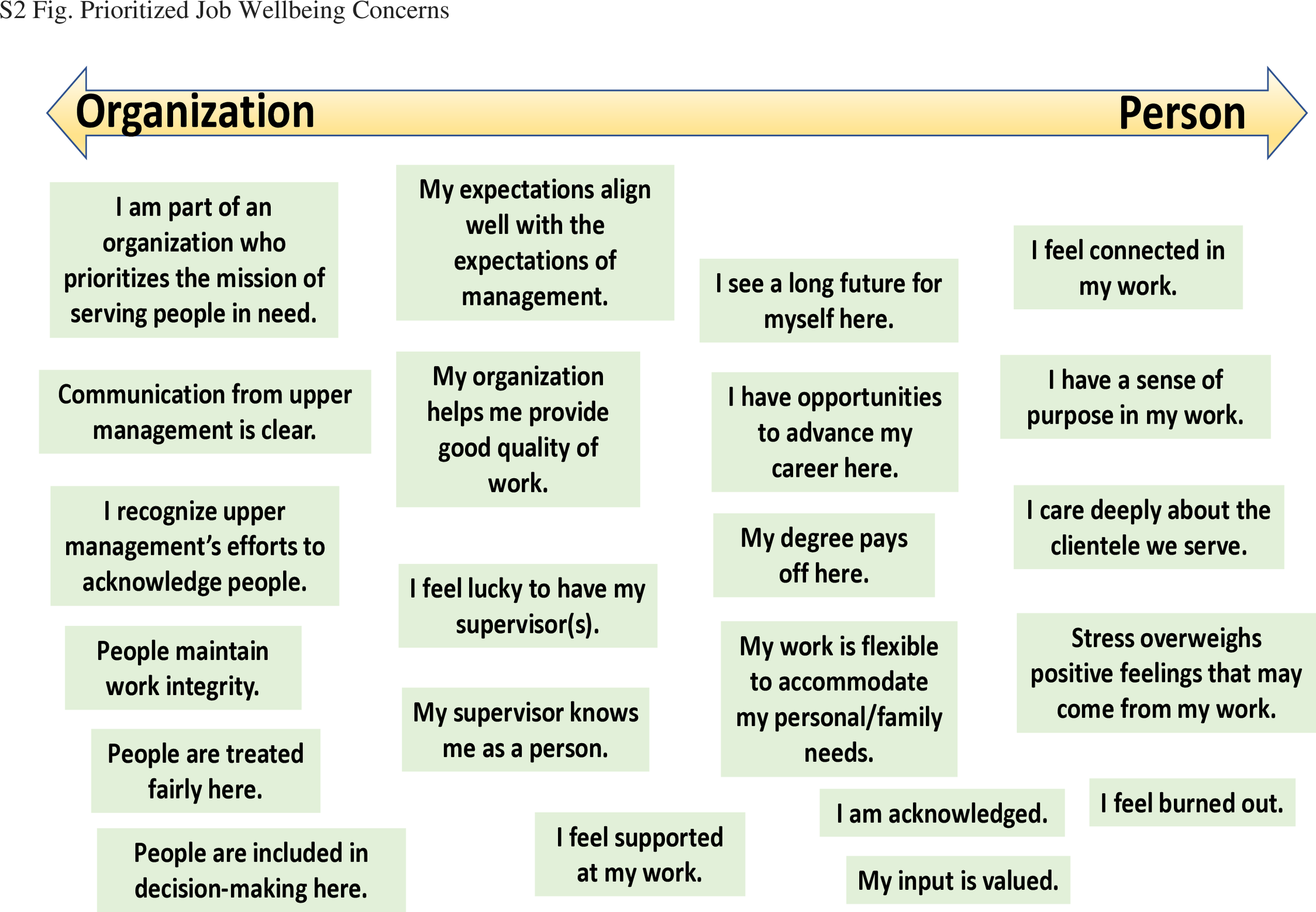

Supplement: S2 Fig — (TIF) [file pone.0321351.s002.tif]
